# Low-dose bortezomib increases the expression of NKG2D and DNAM-1 ligands and enhances induced NK and γδ T cell-mediated lysis in multiple myeloma

**DOI:** 10.18632/oncotarget.13979

**Published:** 2016-12-16

**Authors:** Chao Niu, Haofan Jin, Min Li, Shan Zhu, Lei Zhou, Feng Jin, Yulai Zhou, Dongsheng Xu, Jianting Xu, Lianjing Zhao, Shanshan Hao, Wei Li, Jiuwei Cui

**Affiliations:** ^1^ Cancer Center, The First Hospital of Jilin University, Changchun 130021, China; ^2^ Institute of Translational Medicine, The First Hospital of Jilin University, Changchun 130021, China; ^3^ College of Pharmacy, Jilin University, Changchun 130021, China; ^4^ Department of Hematology, Taian Central Hospital, Taian 271000, China

**Keywords:** bortezomib, multiple myeloma, natural killer cells, gamma-delta T cells

## Abstract

Multiple myeloma (MM) is an incurable hematological malignancy, although bortezomib has markedly improved its outcomes. Growing clinical evidence indicates that enhancing induced natural killer (NK) or γδ T cells for infusion is useful in the treatment of MM. However, whether combination treatment with bortezomib and induced NK and γδ T cells further improves outcomes in MM, and how the treatments should be combined, remain unclear. Herein, we found that low-dose bortezomib did not suppress the viability of induced NK and γδ T cells, but did induce MM cell apoptosis. Importantly, low-dose bortezomib increased the expression of NKG2D and DNAM-1 ligands on MM cells, which sensitized the multiple myeloma cells to lysis by induced NK and γδ T cells. Our results suggested that combination treatment with low-dose bortezomib and induced NK or γδ T cells had a synergistic cytotoxic effect on MM cells. This study provided a proof of principle for the design of future trials and investigation of this combination therapeutic strategy for MM treatment.

## INTRODUCTION

Multiple myeloma (MM) is a hematological malignancy characterized by enhanced proliferation and accumulation of abnormal plasma cells [1, 2]. Over the past two decades, novel agents like the proteasome inhibitor, bortezomib, and the immunomodulatory drug, lenalidomide, have improved clinical outcomes for patients with MM [3–5]. However, curative outcomes remain elusive because of high rates of disease relapse and development of drug resistance [6]. In addition, survival among those in whom lenalidomide and bortezomib fail is especially poor [7]. Therefore, novel and effective treatments are urgently needed. One potential therapeutic option involves the stimulation of the immune system to target and eliminate neoplastic cells.

Innate immune cells, including natural killer (NK) and γδ T cells, play important roles in anti-tumor immune surveillance [8, 9]. Unlike antigen specific T cells, NK and γδ T cells do not require MHC or a specific tumor antigen for target recognition [10, 11]. In clinical trials, immunotherapy using NK or γδ T cells has effectively treated various cancers [9, 12, 13]. Expanded and induced NK and γδ T cells exerted potent specific cytotoxicity against human MM cells *in vitro* [14–16] and the infusion of large numbers of induced NK cells was proven to be a feasible and safe method for MM treatment [17]. In addition, many drugs, such as carfilzomib, lenalidomide, and elotuzumab, enhanced NK cell cytotoxicity against myeloma [18–21]. All of these results suggested that treatment with induced NK and γδ T cells along with chemotherapy drugs provides a promising treatment modality for the eradication of MM cells.

NK and γδ T cell activity was regulated by the balance between the expression levels of numerous inhibitory and activating receptors [22, 23]. Modulation of the ligands to inhibitory and activating receptors on tumor cells represents a promising therapeutic approach that would sensitize cancer cells to γδ T and NK cells and increase cytotoxicity [24, 25]. Interestingly, bortezomib has been shown to decrease the MM cell surface expression of HLA class I (a ligand for killer immunoglobulin-like receptors (KIR), which are inhibitory receptors), thereby sensitizing MM cells to lysis by NK cells isolated from peripheral blood (fresh NK cells) [24]. Our previous study indicated that induced NK cells had much lower KIR expression than did fresh NK cells [26]. Whether bortezomib sensitizes MM cells to lysis by *in vitro* induced NK and γδ T cells, and whether the clinical concentration of bortezomib directly affects the function of NK and γδ T cells remain unknown.

Therefore, in this study, we analyzed the apoptotic effect of various concentrations of bortezomib on MM cells and induced NK and γδ T cells. Furthermore, we investigated whether bortezomib sensitized MM cells to lysis by induced NK and γδ T cells and the mechanism involved in this process. This information may eventually lead to the identification of the optimal dose and regimen for effective therapeutic treatment of MM using bortezomib in combination with immunotherapy using induced NK and γδ T cells.

## RESULTS

### Low-dose bortezomib did not suppress the viability and degranulation of induced NK and γδ T cells

**Figure 1:**
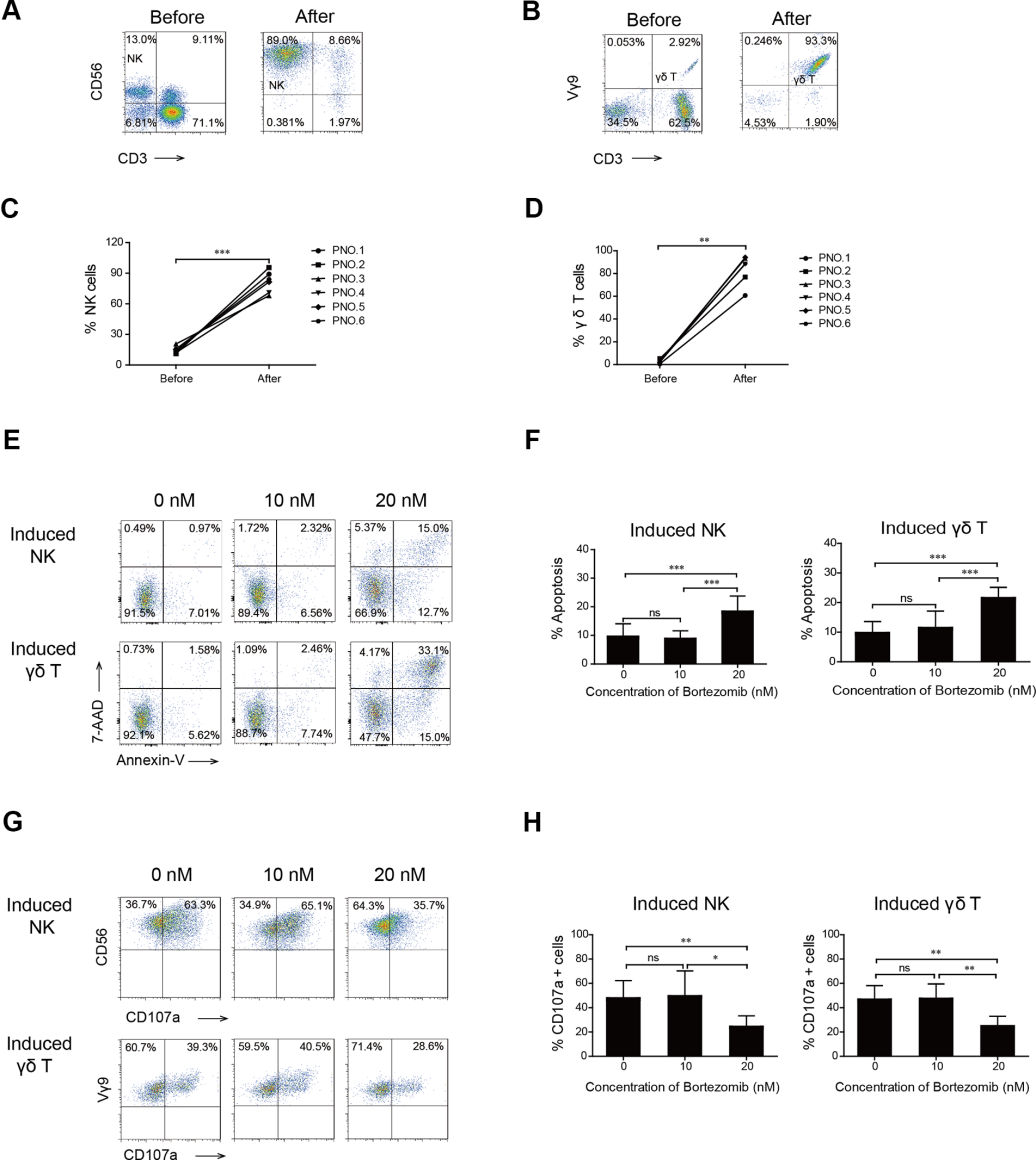
Effects of high- and low-dose bortezomib on the viability and degranulation of induced NK and γδ T cells A representative FACS plot showing the percentage of NK (**A**) and γδ T cells (**B**) cells before and after 14 days of induction in patient number five. Graph showing the percentage of NK (**C**) and γδ T cells (**D**) before and after 14 days of induction in six patients with MM. (**E**) Viability of induced NK and γδ T cells after exposure to bortezomib. One representative experiment is shown. (**F**) Graph showing the apoptosis percentages of induced NK and γδ T cells exposed to increasing doses of bortezomib that were annexin V positive. (**G**) Representative FACS results show CD107a positive cells of induced NK and γδ T cells. (**H**) Comparison of the percentage of CD107a positive cells of induced NK and γδ T cells treated with increasing doses of bortezomib. (*p < 0.05; **p < 0.01; ***p < 0.001; ns: not significant).

The percentage of fresh NK (NK cells in peripheral blood mononuclear cells (PBMCs) before induction) was 15.7% (11.2–20.6%), whereas after 14 days of *in vitro* induction, the percentage of induced NK was 80.2% (67.9–95.6%) (Figure 1A and 1C). Similarly, the percentage of fresh γδ T cells (γδ T cells in PBMCs before induction) was 1.2% (0.51–5.2%), whereas, after induction, the percentage of induced γδ T cells was 79.6% (60.7–93.3%) (Figure 1B and 1D).

Bortezomib at a concentration of 20 nM significantly reduced the percentage, viability, and degranulation of fresh NK and γδ T cells (Figure S1). We also determined whether bortezomib treatment affected the functions of induced NK and γδ T cells. We found that 20 nM bortezomib significantly induced apoptosis of induced NK and γδ T cells. However, 10 nM (low-dose) bortezomib, a concentration higher than that which would be expected 48 h after a dose of intravenous bortezomib (1.0 mg/m^2^), did not suppress the viability of induced NK and γδ T cells (Figure 1E and 1F). This supported the notion that the viability of adoptive transfer of *in vitro* induced NK and γδ T cells 48 h after an intravenous dose of bortezomib would not be inhibited by residual drug.

The expression of CD107a, which represents the killing capacity of immune cells, was determined to evaluate degranulation activity. Our results indicated that low-dose bortezomib did not inhibit the degranulation capacity of induced NK or γδ T cells (Figure 1G and 1H). This provided further evidence that residual drug in the blood did not suppress the killing capacity of *in vitro* induced NK and γδ T cells in bortezomib- treated patients.

### Bortezomib treatment increased the expression of MICA, MICB, Nectin-2, and PVR proteins on MM cells

Bortezomib induced apoptosis in U266 and RPMI-8226 MM cells in a dose-dependent manner (Figure S2). Evaluation of this may be accompanied by unspecific staining during the process of surface marker staining, therefore, we analyzed surface marker expression by gating on PI and annexin V double negative cells in order to exclude apoptotic cells.

**Figure 2:**
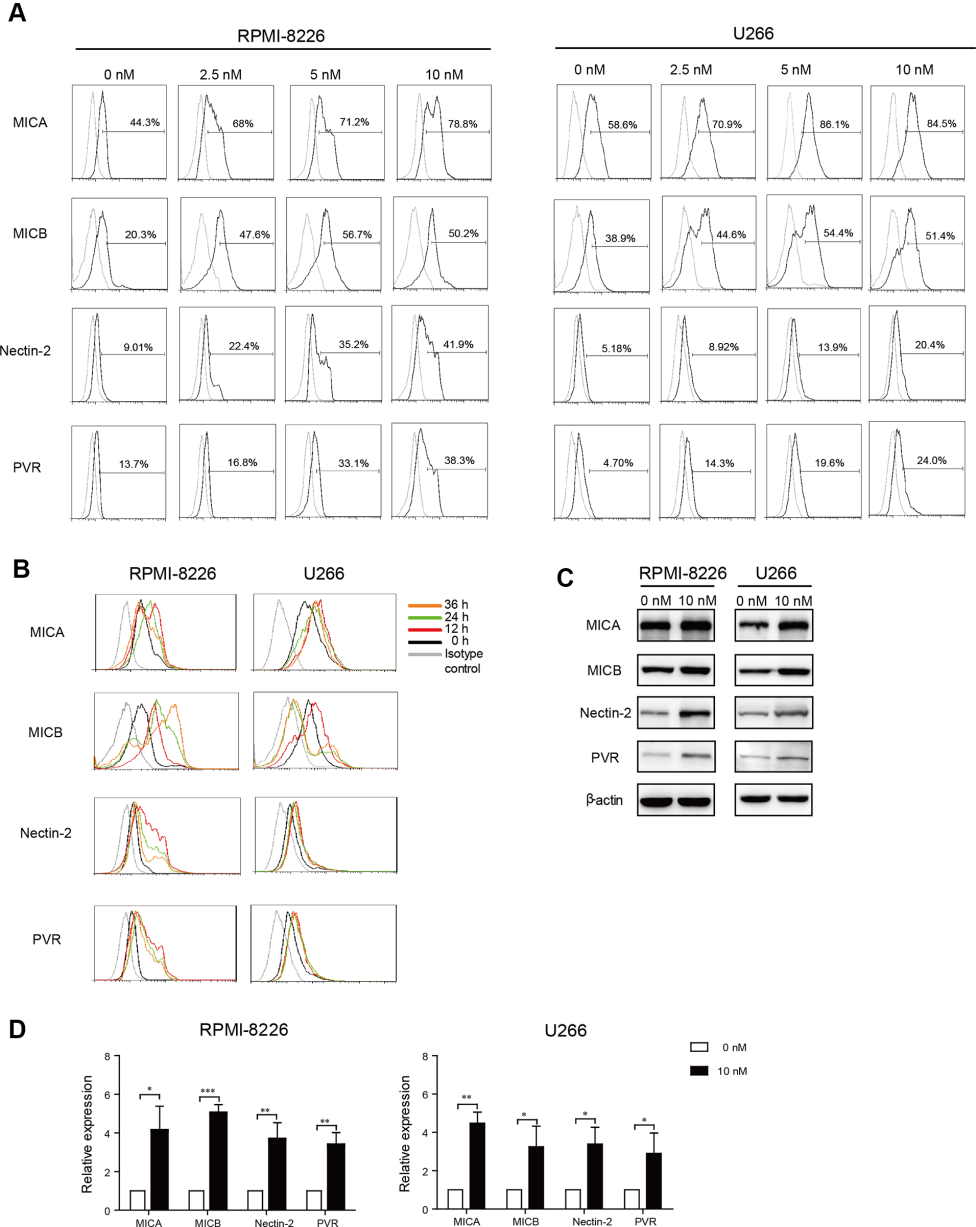
Bortezomib increased MICA, MICB, Nectin-2, and PVR expression on MM cells Annexin V and 7-AAD double negative cells were gated and analyzed for surface marker expression. (**A**) Dose response: RPMI-8226 and U266 cells were exposed to increasing doses of bortezomib for 12 h. (**B**) Time course: RPMI-8226 and U266 cells were incubated with low-dose (10 nM) bortezomib for increasing durations of time. (**C**) Representative western blot analysis of MICA, MICB, Nectin-2, and PVR expression in low-dose bortezomib-treated MM cells. β-actin was used as a loading control of cell lysates. (**D**) qRT-PCR analysis of MICA, MICB, Nectin-2, and PVR expression in RPMI-8226 and U266 cells exposed to low-dose bortezomib for 12 h. Data are expressed as relative expression of MICA, MICB, Nectin-2, and PVR mRNA in low-dose bortezomib-treated MM cells over that of normal saline-treated control cells (*p < 0.05; **p < 0.01; ***p < 0.001).

**Figure 3:**
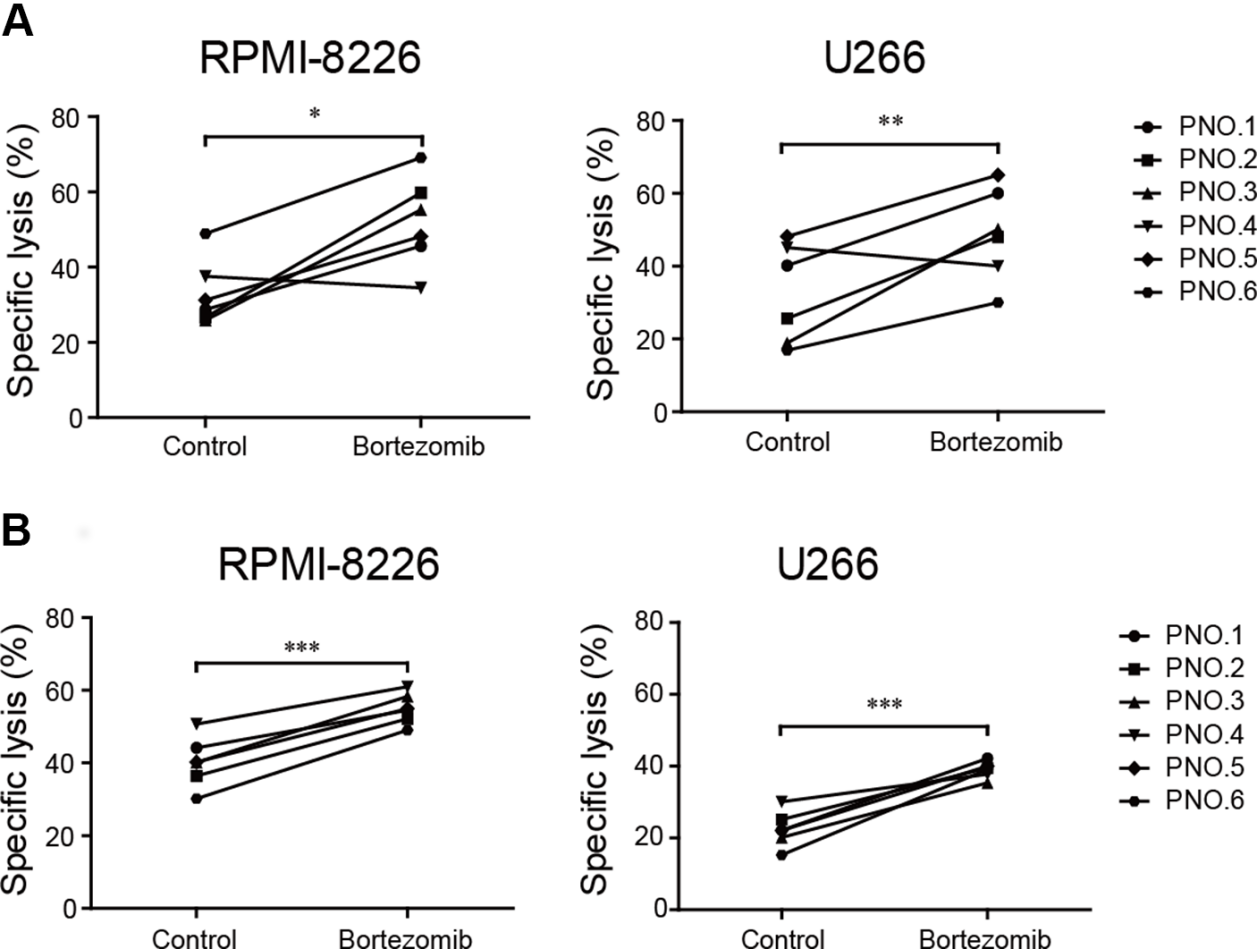
Low-dose bortezomib treatment enhanced the sensitivity of MM cells to induced NK and γδ T cell-mediated lysis (**A**) The cytotoxicity of induced NK cells to low-dose bortezomib-treated and untreated (control) MM cells. (**B**) The cytotoxicity of induced γδ T cells to low-dose bortezomib-treated and untreated (control) MM cells. (*p < 0.05; **p < 0.01; ***p < 0.001).

To further investigate whether bortezomib increased the expression of NKG2D and DNAM-1 ligands in a dose dependent manner, MM cells were treated with varying concentrations of bortezomib. Bortezomib increased the expression of Nectin-2 and PVR on RPMI-8226 and U266 cells and the expression of MICA on RPMI-8226 cells in a dose dependent manner. However, 5 nM bortezomib caused the expression of MICA on U266 cells and MICB on RPMI-8226 and U266 cells to peak (Figure 2A). After incubation with 10 nM bortezomib, the expression of MICA, Nectin-2, and PVR on both RPMI-8226 and U266 cells and MICB on U266 cells reached a peak at 12 h (Figure 3B). In addition, the expression of MICB on RPMI-8226 cells consistently increased with time (Figure 2B). Increases in NKG2D and DNAM-1 ligand expression on MM cells were further confirmed by western blot (Figure 2C).

To further validate the expression of MICA, MICB, Nectin-2, and PVR, the mRNA levels of these genes were analyzed. Total RNA was isolated from RPMI-8226 and U266 cells incubated with 10 nM bortezomib for 12 h. Based on quantitative real-time reverse transcription PCR (qRT-PCR) analysis of the treated cells, levels of MICA, MICB, Nectin-2, and PVR mRNA were significantly greater in MM cells incubated with 10 nM bortezomib compared to those in control MM cells (0 nM) (Figure 2D).

### Low-dose bortezomib sensitized MM cells to lysis by induced NK and γδ T cells *in vitro*

We have shown that low-dose bortezomib increased MICA, MICB, PVR, and Nectin-2 expression on MM cell lines. Because increased MICA, MICB, PVR, and Nectin-2 expression is associated with increased sensitivity of MM cells to NK and γδ T cell-mediated lysis, we evaluated whether bortezomib treatment augmented the sensitivity of MM cells to induced NK and γδ T cell-mediated cytolysis *in vitro*. Compared with untreated controls, exposure to bortezomib significantly enhanced the sensitivity of MM cells to lysis by NK cells (Figure 3A) and γδ T cells (Figure 3B) induced from the PBMCs of patients with MM. This supported the notion that the cytotoxicity of adoptively transferred induced NK and γδ T cells to MM cells was enhanced by residual bortezomib in patients with MM.

### Induced NK and γδ T cells displayed a higher level of NKG2D and DNAM-1 expression

**Figure 4:**
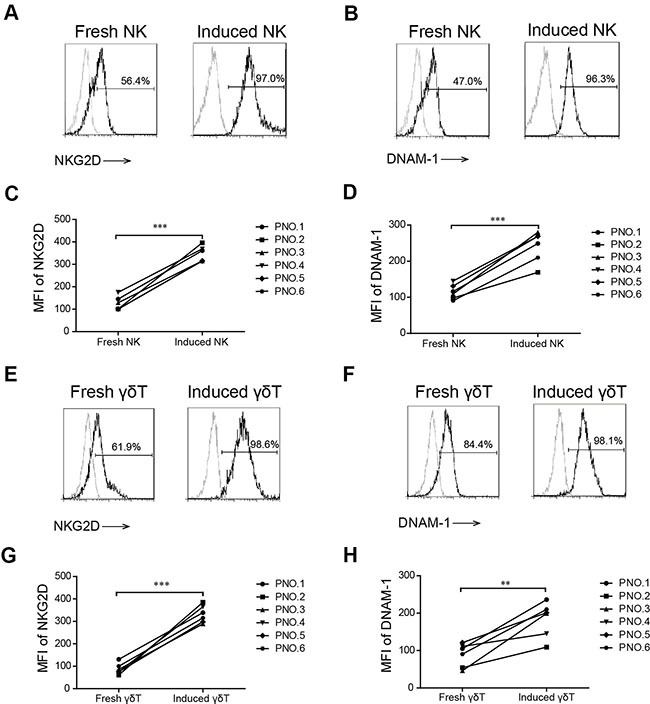
NKG2D and DNAM-1 expression in induced and fresh NK and γδ T cells The percentage of NKG2D (**A**) and DNAM-1 (**B**) on fresh and induced NK cells in patient number five. The mean fluorescence index (MFI) of NKG2D (**C**) and DNAM-1 (**D**) on induced and fresh NK cells in six patients with MM. The percentage of NKG2D (**E**) and DNAM-1 (**F**) on fresh and induced γδ T cells in patient number five. The MFI of NKG2D (**G**) and DNAM-1 (**H**) on induced and fresh γδ T cells in six patients with MM. (**p < 0.01; ***p < 0.001).

NKG2D and DNAM-1 are the receptors of MICA, MICB, Nectin-2, and PVR that are important for the cytotoxicity function of both NK and γδ T cells. We showed that low-dose bortezomib increased MICA, MICB, PVR, and Nectin-2 expression on MM cells as the target cells. We also evaluated whether effector cells expressed NKG2D and DNAM-1 and found that the expressions of NKG2D and DNAM-1 on induced NK and γδ T cells were significantly higher than those on fresh NK and fresh γδ T cells (Figure 4). The data in Figure 4A,4B,4E, and 4F represent one representative patient and the data in Figure 4C,4D,4G,and 4H represent all six patients. Overall, these results indicated that induced NK and γδ T cells were strongly activated immune cells that were more cytotoxic to MM cells when expressing NKG2D and DNAM-1 ligands.

### Low-dose bortezomib sensitized MM cells to lysis by induced NK and γδ T cells through NKG2D and DNAM-1 ligands

**Figure 5:**
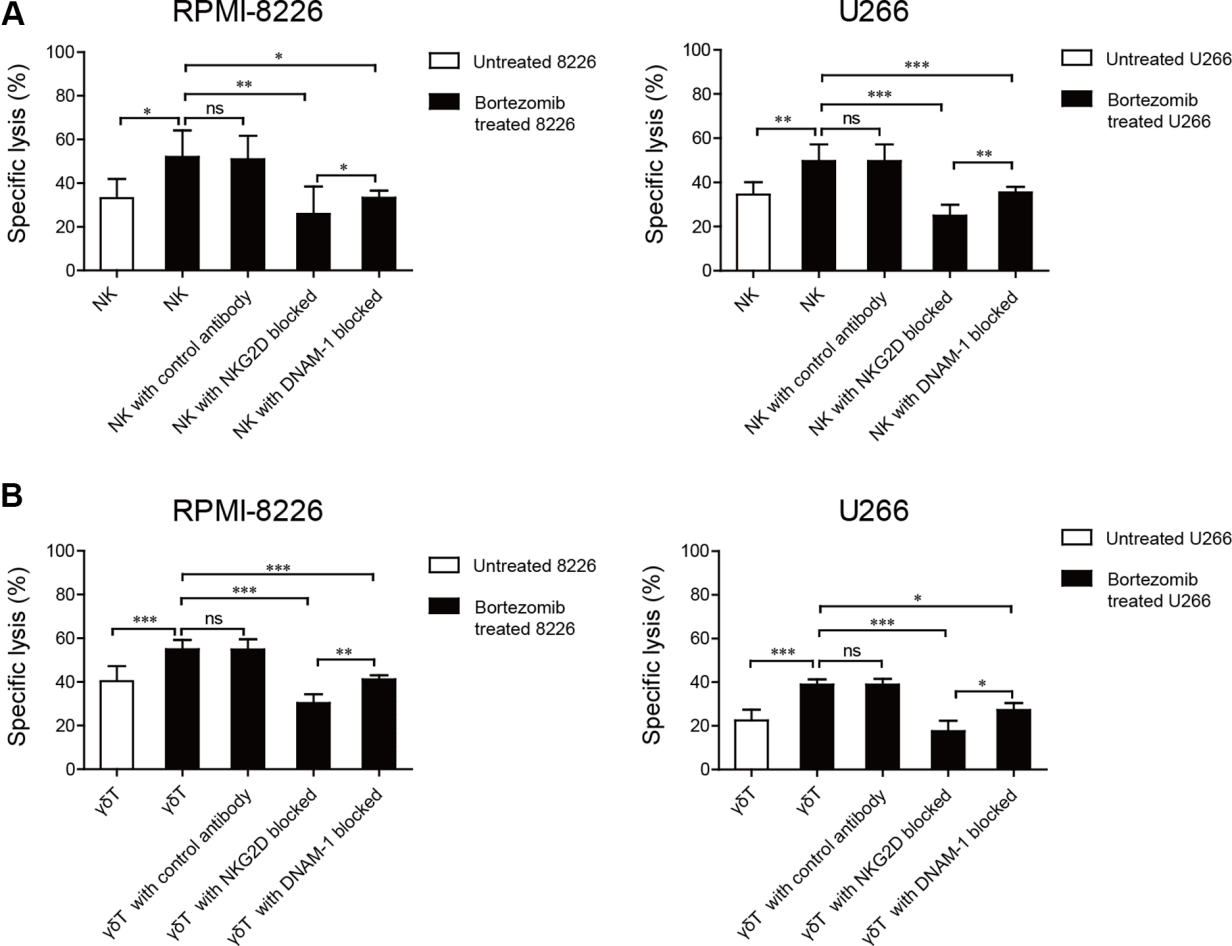
Enhanced NK and γδ T cell cytotoxicity against low-dose bortezomib-treated MM cells correlated with the interactions between NKG2D and DNAM-1 receptors and their ligands Increased NK cell and γδ T cell killing was abolished by blocking NKG2D or DNAM-1 on NK cells (**A**) and γδ T cells (**B**). (*p < 0.05; **p < 0.01; ***p < 0.001; ns: not significant).

We observed that there was a higher level of MICA, MICB, PVR, and Nectin-2 expression on bortezomib-incubated MM cells than on unincubated MM cells, and a high level of NKG2D and DNAM-1 expression on induced NK and γδ T cells. We next evaluated whether this amount of NKG2D and DNAM-1 ligand upregulation was relevant to the increased sensitivity of MM cells to induced NK and γδ T cell-mediated lysis. Induced NK and γδ T cells were incubated with NKG2D or DNAM-1 blocking antibody before cytotoxicity assays were performed with low-dose bortezomib-treated MM cells. We observed that NKG2D and DNAM-1 blocking antibodies considerably reduced NK and γδ T cell cytotoxicity to low-dose bortezomib-treated MM cells, whereas a control monoclonal antibody (mAb) did not decrease the cytotoxicity. This indicated that the increased cytotoxicity of induced NK and γδ T cells was dependent on NKG2D and DNAM-1 activation. Moreover, it appeared that the cytotoxicity of induced NK and γδ T cells against low-dose bortezomib-treated MM cells was more dependent on NKG2D recognition than DNAM-1 because anti-NKG2D mAbs more substantially reduced the cytotoxicity (Figure 5A and 5B).

## DISCUSSION

Recent efforts to combine conventional therapy with immunotherapy for MM have resulted in the development of several promising therapies. However, questions about how to combine and sequence different therapies for the treatment of MM remain unanswered. In this study, we found that high dose (20 nM) bortezomib induced the apoptosis of induced NK and γδ T cells and thereby inhibited NK and γδ T cell-mediated cytotoxicity, whereas low-dose (10 nM) bortezomib enhanced MM cell sensitivity to induced NK and γδ T cell-mediated lysis and supported NK and γδ T cell-based immunotherapy. Therefore, the optimal clinical dose and regimen for bortezomib administration must be established. Typically, bortezomib is administered twice weekly for two weeks, with a minimum of 72 h between doses, to allow for the restoration of proteasome function in normal cells. Each cycle of four doses (i.e., administration on days 1, 4, 8, and 11) is followed by a 10-day rest [27]. A pharmacokinetic study indicated that 24–48 h after the day 11 dose, the concentration of bortezomib in the blood was approximately 10 nM [28]. Similarly, we confirmed that 10 nM bortezomib increased the expression of NKG2D and DNAM-1 ligands on MM cells and had little effect on the viability of fresh (Figure S1) and induced NK and γδ T cells *in vitro*. In contrast, concentrations of bortezomib immediately following infusion were high enough to not only eliminate MM cells, but also have unfavorable effects on immunocytes. Therefore, an infusion of expanded and activated immunocytes to patients within the time period when the residual MM cells remain sensitized to NK or γδ T cells and the concentration of bortezomib has fewer unfavorable effects on immunocytes may represent an effective cellular immunotherapy regimen. The blood of patients can be drawn one or two days before treatment, then it would take about two weeks *ex vivo* to induce and expand sufficient numbers of immunocytes for infusion. As a result, adaptive infusion of induced NK and γδ T cells may increase the therapeutic effects of conventional bortezomib therapy.

Over the last decade, we have witnessed a growing interest in the anti-tumor function of innate immune cells, such as NK and γδ T cells, that have shown therapeutic potential in patients with MM [15, 17]. Furthermore, γδ T cells and NK cells seem to have synergistic effects. The γδ T cells induce robust NK cell-mediated antitumor cytotoxicity [29] and NK cells maintain the homeostasis of γδ T cells [30]. In addition, γδ T cells have been shown to kill NK-resistant tumor cells, indicating a potential for synergistic therapeutic effects. Nevertheless, patients with MM have fewer or less active NK and γδ T cells [31–33]. In our study, we successfully expanded NK and γδ T cells from patients with MM and found no difference in the expansion fold of NK and γδ T cells between patients with MM and normal healthy controls (Figure S3). Induced NK and γδ T cells are strongly activated immune cells that have a greater expression of CD69 than fresh NK and γδ T cells, as indicated by our previous study [26]. Therefore, treatment with adoptively transferred, *in vitro* expanded and induced NK and γδ T cells might be a prospective therapeutic method for patients with MM.

Despite the link between malignant transformation and expression of danger signals, tumor cells are often insufficiently recognized by NK and γδ T cells. This may limit the ability of both endogenous and adoptively infused autologous NK and γδ T cells to induce antitumor effects. Therefore, increasing the surface density of stimulatory ligands represents an attractive approach to elicit or enhance NK or γδ T cell-based antitumor responses. The anti-cancer properties of bortezomib are complex and are mediated in part by interfering with host immunity [34]. In our study, we observed that bortezomib not only induced the apoptosis of MM cells, but also enhanced the expression of activating ligands, including MICA, MICB, PVR, and Nectin-2. Bortezomib also increased Fas and TRAIL ligand expression (Figure S4), thereby enhancing the cytotoxic function of immune cells [35, 36]. Therefore, bortezomib activated immune responses through multiple mechanisms. However, low-dose bortezomib enhanced NK and γδ T cell-mediated lysis of MM cells primarily by upregulating the expression of NKG2D and DNAM-1 ligands on MM cells.

In conclusion, treatment with the combination of low-dose bortezomib and induced NK or γδ T cells had a synergistic cytotoxic effect on MM cells. The mechanism underlying the enhanced tumor cell killing may involve the increased expression of MICA, MICB, PVR, and Nectin-2 proteins on MM cells and the ligands of the activating receptors, NKG2D and DNAM-1. The results of this study provided a proof of principle for the design of future trials and investigation of this combination therapeutic strategy in clinical settings.

## MATERIALS AND METHODS

### Patient enrollment

Six patients newly diagnosed with MM were enrolled in this study, all of whom provided written informed consent for the use of the biospecimens for research purposes. The study was carried out in accordance with the approved guidelines “Use of experimental animals and human subjects”. The study was approved by the Ethics Committee of the First Hospital of Jilin University. Patient information is shown in Table 1.

**Table 1:** Patient clinical characteristics

Patients No.	Gender	Age	Type of Tumor	Stages of Disease
1	Male	68	MM	ISS III/DS IIIA
2	Female	59	MM	ISS I/DS IIA
3	Male	59	MM	ISS I/DS IIIB
4	Male	46	MM	ISS I/DS IIA
5	Male	57	MM	ISS III/DS IIA
6	Female	64	MM	ISS III/DS IIIA

### Pharmacological agents

Bortezomib was purchased from Xian Janssen Pharmaceutical Ltd. (Xi’an, China) and dissolved in normal saline to a concentration of 100 nM.

### Cell culture

The MM cell lines, U266 and RPMI-8226, were kindly provided by Dr. Jifan Hu at Stanford University Medical School, Palo Alto Veterans Institute for Research, Palo Alto, CA, USA. Both cell lines were cultured in RPMI-1640 medium (Gibco, Grand Island, NY, USA) supplemented with 10% heat-inactivated FBS (Gibco), 100 U/mL penicillin, and 100 mg/mL streptomycin (Gibco) at 37°C in a humidified 5% CO_2_ incubator.

### Expansion and isolation of induced NK and γδ T cells

Heparinized peripheral blood samples were obtained from the six patients with MM. Blood samples were centrifuged at 1800 × g for 10 min and plasma was transferred to new tubes. PBMCs were isolated by density gradient centrifugation using Ficoll medium (Axis-Shield PoC AS, Oslo, Norway) at 800 × g for 30 min. To obtain induced NK cells, PBMCs at a density of 1 × 10^6^ cells/mL were cultured in AlyS 505 NK-EX (CSTI, Sendai-shi, Miyagi, Japan) containing 5% auto-plasma, 500 U/mL interleukin (IL)-2 (Miltenyi Biotec, Bergisch Gladbach, Germany), and 10 ng/mL anti-CD3 mAbs (Miltenyi Biotec). To amplify γδ T cells, PBMCs were cultured in complete medium with 1 μM zoledronate (zoledronic acid, Jilin Province Xidian Pharmaceutical Sci-Tech Development Co., Jilin, China) and 400 U/mL IL-2. Fresh complete medium supplemented with IL-2 (500 U/mL) was added every two or three days. Purified induced CD3-CD56+ NK cells and CD3+Vγ9+ γδ T cells were isolated using a flexible BD Influx™ cell sorter (BD Biosciences, San Jose, CA, USA).

### *In vitro* treatment and flow cytometry

MM cells were incubated with 0, 5, 10, and 20 nM bortezomib for 12 h and MM cells were incubated with 10 nM bortezomib for 12, 24, and 36 h. Cells were then stained with Nectin-2-PE (BD Biosciences), PVR-PE, MICA-PE, and MICB-APC (R&D Systems, Minneapolis, MN, USA). Purified induced NK and γδ T cells were stained with CD56-FITC (BD Biosciences) or Vγ9-FITC, CD3-PerCp (BD Biosciences), NKG2D-APC (BD Biosciences), and DNAM-1-PE (BD Biosciences). Appropriate isotype-matched antibodies were used as controls. Data were analyzed using a BD FACSCalibur (BD Biosciences) with Cell Quest Pro software and final analysis was performed using FlowJo software (Tree Star Inc., Ashland, OR, USA).

### Detection of apoptosis

MM cells were incubated with 0, 5, 10, and 20 nM bortezomib for 12 h. In addition, induced NK and γδ T cells were treated with 0, 10, and 20 nM bortezomib for 12 h. The proportions of living, dead, and apoptotic cells were determined using an annexin V and 7-AAD staining kit (eBioscience, San Diego, CA, USA) according to the manufacturer's protocol. Quantitative analysis was performed using flow cytometry.

### Degranulation assay

A CD107a translocation assay was used to evaluate the degranulation activity of the fresh and induced NK and γδ T cells as previously described [37]. Purified induced NK and γδ T cells from patients with MM were incubated with 0, 10, and 20 nM bortezomib for 12 h. The drug-treated cells were mixed with RPMI-8226 cells at a ratio of 10:1 and incubated at 37°C in 5% CO_2_. After 4 h of incubation, the cells were collected and stained with CD56-FITC or Vγ9-FITC, CD3-PerCp, and CD107a-APC (BD Biosciences). After staining, the cells were analyzed using a BD FACSCalibur flow cytometer (BD Biosciences).

### Quantitative real-time reverse transcription PCR (qRT-PCR)

Total RNA was extracted from MM cells treated with 10 nM bortezomib using a Promega SV Total RNA Isolation System (Promega Corporation, Madison, WI, USA), according to the manufacturer's instructions. After removing genomic DNA contamination with DNase I (Sigma, St. Louis, MO, USA), M-MLV reverse transcriptase (Invitrogen, Carlsbad, CA, USA) was used to synthesize cDNA. MICA, MICB, Nectin-2, and PVR mRNA expression was quantified by qRT-PCR using a CFX384^TM^ Real Time system (Bio-Rad, Hercules, CA, USA). The PCR reactions were set up in a final volume of 10 μL with 5 μL SYBR Green qPCR Mix (Roche, Indianapolis, IN, USA) and 10 pmol of each sense and antisense primer. Primer sequences were as follows: for human MICA gene, sense: 5′-GTTTCTGCTGTTGCTGCT GCTGC-3′ and anti-sense: 5′-ATCCCTGTGGTCACTC GTCC-3′; for human MICB gene, sense: 5′-ATGTTTCT GCTGCTATGCCATG-3′ and anti-sense: 5′-GACCCTCT GCCGCTGATGT-3′; for human PVR gene, sense: 5′-TGA GGATGTTCGGGTTGCG-3′ and anti-sense: 5′-CTGGCT CGTATTGGGCATC-3′; for human Nectin-2 gene, sense: 5′-ACCTGCGAACCACCAGAATG-3′ and anti-sense: 5′-CTTGCCCAGTGCTCTGCTT-3′. The mRNA of human β-actin was also quantified and used as an internal standard. Primers for β-actin were as follows: sense: 5′-AAGATC ATTGCTCCTCCTG-3′ and antisense: 5′-CGTCATACTC CTGCTTGCTG-3′. The PCR amplification procedure was as follows: 10 s at 95°C followed by 40 cycles of 5 s at 95°C and 30 s at 64°C. Each standard and sample value was determined in triplicate in three independent experiments. In each experiment, expression of MICA, MICB, Nectin-2, and PVR under each experimental condition was calculated using threshold cycle (Ct) values standardized to β-actin (housekeeping gene), applying the 2-(ΔCt) method [38].

### Western blot analysis

Protein samples from MM cells treated with 10 nM bortezomib were homogenized using radio-immunoprecipitation assay (RIPA) lysis buffer. Equal amounts of protein were separated from each sample using SDS-PAGE and transferred onto polyvinylidene fluoride (PVDF) membranes. The membranes were then incubated for 1 h with blocking buffer containing 5% skimmed milk and incubated with the primary antibodies for MICA, MICB, Nectin-2, and PVR along with rabbit anti-goat or goat anti-mouse IgG antibody (R&D Systems). An Enhanced Chemiluminescence (ECL) Detection System was used to visualize the proteins.

### Cytotoxicity assay

A calcein-acetoxymethyl (AM) release assay was used as previously described to assess cytotoxicity [39]. The target MM cells were pretreated with low-dose (10 nM) bortezomib for 12 h, labeled with 3.5 μM of calcein-AM, and incubated in a humidified incubator at 37°C with 5% CO_2_ for 30 min. After washing twice with PBS, target cells were adjusted to a concentration of 5 × 10^4^ cells/mL with 5% FBS RPMI-1640 medium and seeded into 96-well plates. As to the experimental group, the induced NK and γδ T effector cells (100 μL), treated and untreated with antibodies to NKG2D or DNAM-1 (R&D Systems), were added to the target cells at a ratio of 10:1 and incubated at 37°C for 4 h. During the assay, spontaneous release controls were set by incubating target cells with low-dose bortezomib in medium alone and maximum release controls were set when target cells were treated with 1% Triton X-100. After incubation, the supernatant was harvested and transferred to a new plate. Fluorescence at 485 nm of excitation light wavelength and 528 nm of emission wavelength was assessed using a BioTek Synergy HT Microplate Reader (BioTek Instruments, Winooski, VT, USA). Specific lysis was calculated according to the following formula: [(experimental release – spontaneous release) / (maximum release – spontaneous release)] × 100%. All experiments were performed in triplicate and at least three independent experiments were completed.

### Statistical analysis

Data were analyzed using non-parametric tests (Wilcoxon's signed rank test, Wilcoxon's sum-rank test) using SPSS 19.0 software (SPSS Inc., Chicago, IL, USA). The comparative *C*T method was applied for the qRT-PCR assay according to the delta-delta *C*T method [40]. Results were indicated as mean ± SD and considered significant at a *p* < 0.05.

### Authors’ contributions

J.C., W.L., Y.Z., S.Z. and H.J. conceived and designed the research. C.N., M.L., F.J., S.H., L-J. Z., and L.Z. performed the experiments and analyzed the data. J.X. and D.X. contributed to sample collection. C.N. and S.Z. wrote the paper. All authors reviewed the manuscript.

## SUPPLEMENTARY MATERIALS FIGURES


