# Supplementary material for: Low-dose bortezomib increases the expression of NKG2D and DNAM-1 ligands and enhances induced NK and γδ T cell-mediated lysis in multiple myeloma
Source: Oncotarget. 2016 Dec 16;8(4):5954–64. doi: 10.18632/oncotarget.13979 (PMC5351604; doi:10.18632/oncotarget.13979)
Supplement: Supplementary file 1 [file oncotarget-08-5954-s001.pdf]

Low-dose bortezomib increases the expression of NKG2D and DNAM-1 ligands and enhances induced NK and γδ T cell-mediated lysis in multiple myeloma

Supplementary Materials

Supplementary methods

To evaluate the effects of high- and low-dose bortezomib on the percentage and viability of fresh NK and γδ T cells, PBMCs from the blood of patients with MM were treated with 0, 5, 10, and 20 nM bortezomib for 12 h. Thereafter, cells were stained with CD3-PerCP, CD56-FITC, or Vγ9-FITC. The proportions of living, dead, and apoptotic cells were determined using an annexin V and 7-AAD staining kit. For the degranulation assay, PBMCs from the blood of patients with MM were treated with 0, 10, and 20 nM bortezomib for 12 h. The drug-treated cells were mixed with RPMI-8226 cells at a ratio of 10:1 and incubated at 37°C in 5% CO_2. After 4 h of incubation, the cells were collected and stained with CD56-FITC or Vγ9-FITC, CD3-PerCp, and CD107a-APC. Quantitative analysis was performed by flow cytometry.

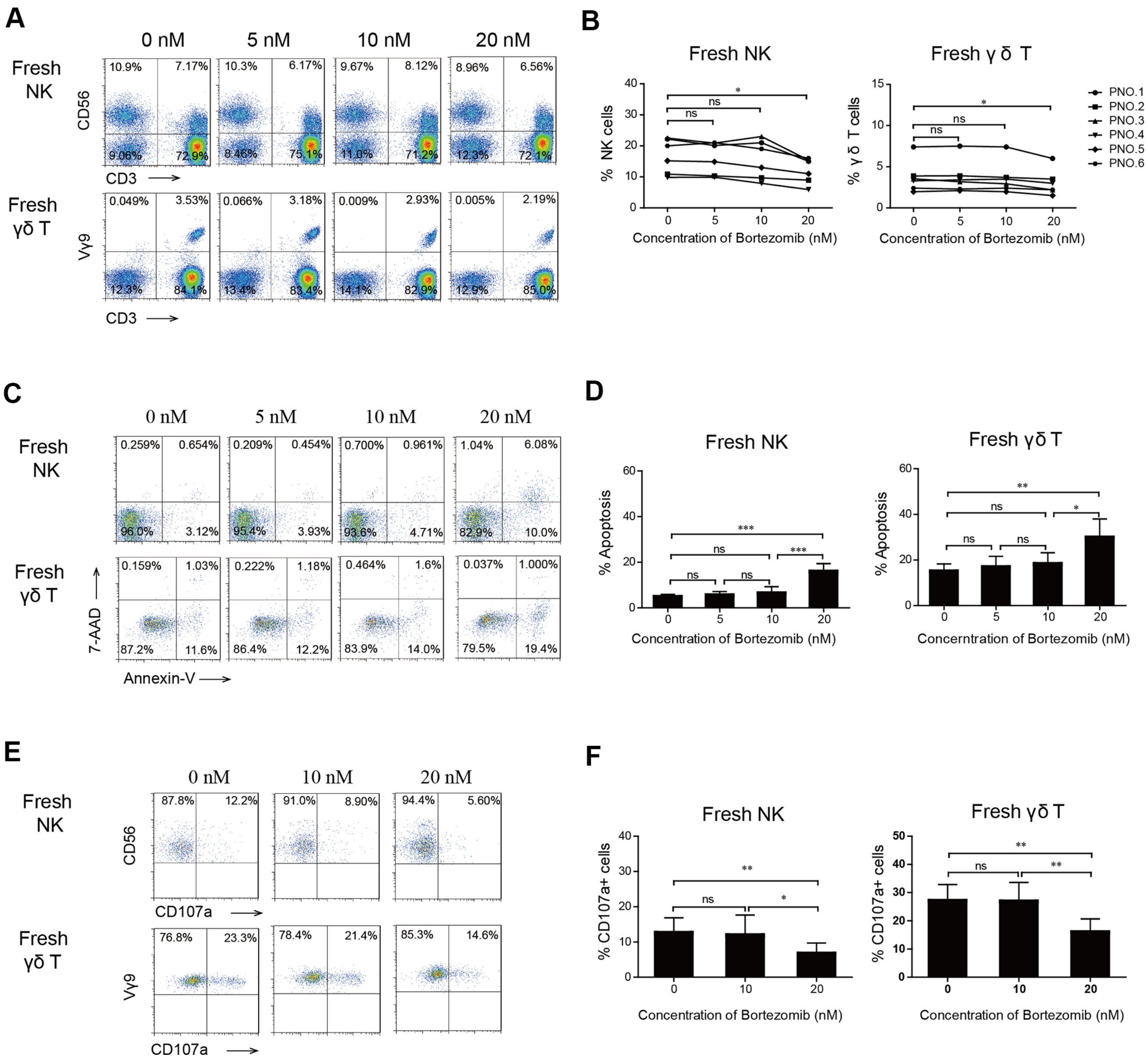

Supplementary Figure S1: Effect of high- and low-dose bortezomib on the percentage, viability, and degranulation of fresh NK and γδ T cells. (**A**) Representative FACS plots showing the percentage of fresh NK and γδ T cells in drug-treated PBMCs from one representative patient. (**B**) Graph showing the percentage of NK and γδ T cells in drug-treated PBMCs from the six patients with MM. (**C**) Viability of fresh NK and γδ T cells after exposure to bortezomib. One representative experiment is shown. (**D**) Apoptosis percentage of fresh NK and γδ T cells that were annexin V positive. (**E**) Representative FACS results indicated CD107a positive cells in fresh NK and γδ T cells in drug-treated PBMCs from one patient. (**F**) Comparison of CD107a positive cells in fresh NK and γδ T cells in drug-treated PBMCs from the six patients with MM. (**p* < 0.05; ***p* < 0.01; ****p* < 0.001; ns: not significant).

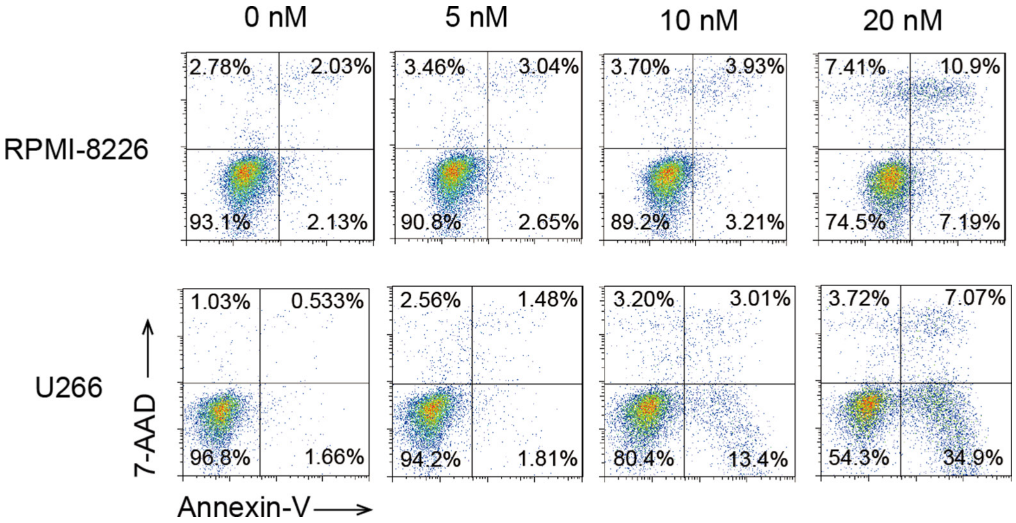

Supplementary Figure S2: Bortezomib induced MM cells apoptosis in a dose dependent manner. MM cells were incubated with various doses of bortezomib for the indicated times. The data represent one of three individual experiments.

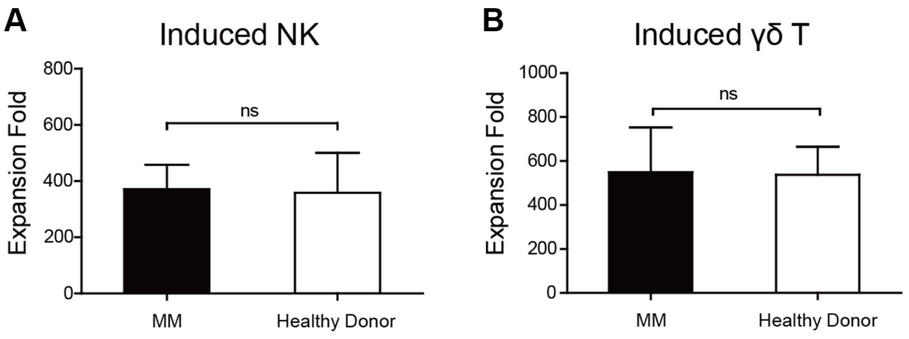

Supplementary Figure S3: Expansion potential of NK and γδ T cells from patients with MM and healthy donors. NK and γδ T cells were induced from the PBMCs of six patients with MM and six healthy donors. After 14 days of induction, the expansion potential of NK and γδ T cells was calculated by dividing the absolute number of NK or γδ T cells after 14 days of culture by their number on day 0. The absolute output number of these two types of immune cells was calculated by multiplying the total number of viable cells by the percentages of the two immune cells, as determined by flow cytometry (ns: not significant).

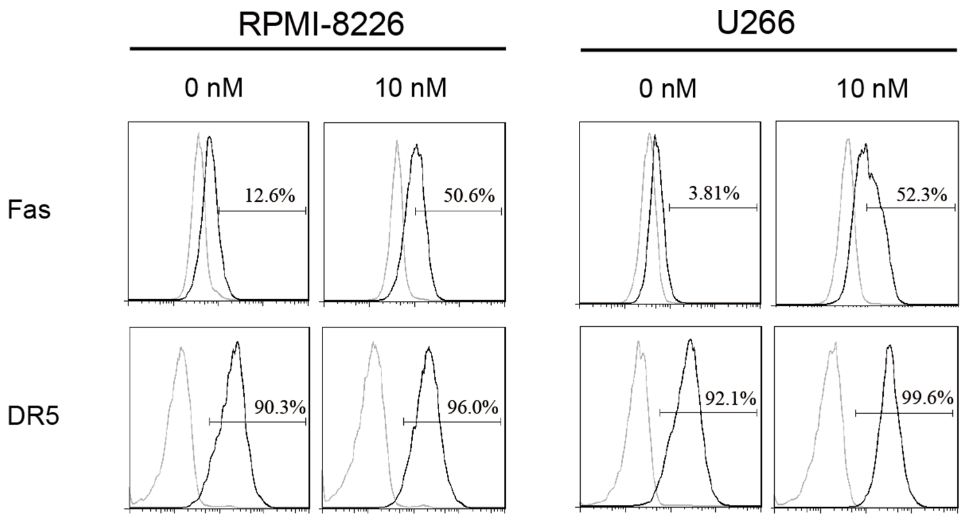

Supplementary Figure S4: Low-dose bortezomib increased Fas and DR5 expression on MM cells. RPMI-8226 and U266 cells were exposed to low-dose bortezomib for 12 h. The expression of Fas and DR5 was detected by flow cytometry.
